# Optimum and Decorrelated Constrained Multistage Linear Phenotypic Selection Indices Theory

**DOI:** 10.2135/cropsci2019.04.0241

**Published:** 2019-10-31

**Authors:** J. Jesus Cerón-Rojas, Fernando H. Toledo, Jose Crossa

**Affiliations:** Biometrics and Statistics Unit, International Maize and Wheat Improvement Center (CIMMYT), Apdo. Postal 6-641, 06600, Mexico City, Mexico

## Abstract

Some authors have evaluated the unconstrained *optimum* and *decorrelated* multistage linear phenotypic selection indices (OMLPSI and DMLPSI, respectively) theory. We extended this index theory to the constrained multistage linear phenotypic selection index context, where we denoted OMLPSI and DMLPSI as OCMLPSI and DCMLPSI, respectively. The OCMLPSI (DCMLPSI) is the most general multistage index and includes the OMLPSI (DMLPSI) as a particular case. The OCMLPSI (DCMLPSI) predicts the individual net genetic merit at different individual ages and allows imposing constraints on the genetic gains to make some traits change their mean values based on a predetermined level, while the rest of them remain without restrictions. The OCMLPSI takes into consideration the index correlation values among stages, whereas the DCMLPSI imposes the restriction that the index correlation values among stages be null. The criteria to evaluate OCMLPSI efficiency vs. DCMLPSI efficiency were that the total response of each index must be lower than or equal to the single-stage constrained linear phenotypic selection index response and that the expected genetic gain per trait values should be similar to the constraints imposed by the breeder. We used one real and one simulated dataset to validate the efficiency of the indices. The results indicated that OCMLPSI accuracy when predicting the selection response and expected genetic gain per trait was higher than DCMLPSI accuracy when predicting them. Thus, breeders should use the OCMLPSI when making a phenotypic selection.

In a two-stage context, Ceron-Rojas et al. (2019) described and evaluated the unconstrained *optimum and decorrelated* multistage linear phenotypic selection indices (OMLPSI and DMLPSI, respectively) theory and concluded that OMLPSI efficiency when predicting the net genetic merit was higher than the DMLPSI efficiency and that breeders should use the OMLPSI when making phenotypic selection. The main difference between the two indices is that although the OMLPSI takes into consideration the correlation values among stages when predicting the net genetic me it, the DMLPSI imposes the restriction that the correlation values among stages be null when it makes the prediction. The main characteristic of the OMLPSI (DMLPSI) in a two-stage context is that at Stage 1, OMLPSI (DMLPSI) is a partial index, but at Stage 2, it is a complete index. This selection procedure is called the part and whole index selection method (Young, 1964; Saxton, 1983) and is valid for any number of stages. The OMLPSI (DMLPSI) is more efficient than the independent culling method because it uses all available information at each stage and incorporates the genetic correlations between traits in the prediction.

The OMLPSI (DMLPSI) combines the single-stage linear phenotypic selection index (LPSI) theory (Smith, 1936; Hazel, 1943) with the independent culling selection method (Cochran, 1951; Young, 1964; Cunningham, 1975; Xu and Muir, 1992) and is useful for selecting more than one trait in the multistage selection context. Breeders apply the OMLPSI (DMLPSI) mainly in animal and tree breeding where, due to early culling, OMLPSI (DMLPSI) is a cost-saving strategy for improving several traits because they do not need to measure all traits at each stage. The OMLPSI (DMLPSI) increases selection intensity on traits measured at an earlier age, and, with fixed facilities, this index selects a greater number of individuals at an earlier age (Xu and Muir, 1991, 1992).

The OMLPSI values may have a non-normal distribution after the first selection stage, and to derive selection intensities for more than two stages, this index requires numeric multiple integration techniques. To solve this problem, the DMLPSI minimizes the mean squared difference between the index and the net genetic merit at each stage under the restriction that the covariance between the DMLPSI values at different stages be zero, thus preventing the correlation between DMLPSI values at different stages. Under this restriction, truncation points and selection intensities can be determined for a fixed total proportion before the breeder carries out selection, and the selected individual index values after the first selection stage may be normally distributed (Xu and Muir, 1992). Nevertheless, due to the indicated restriction, the DMLPSI selection response and accuracy after the first stage could be lower than the OMLPSI selection response.

One additional problem with the OMLPSI (DMLPSI) expected genetic gain per trait (or multitrait selection response) is that its values can increase or decrease in a positive or negative direction without control. In the single-stage context, Kempthorne and Nordskog (1959) developed the restricted LPSI that allows imposing restrictions equal to zero on the expected genetic gain of some traits. Other authors (Mallard, 1972; Harville, 1975; Tallis, 1985) extended the Kempthorne and Nordskog (1959) approach and developed a single-stage constrained LPSI (SCLPSI) that attempts to make some traits change their expected genetic gain values based on a predetermined level while the rest of the traits remain without restrictions. Itoh and Yamada (1987) showed that in reality there is only one optimum SCLPSI; that is, the Mallard (1972), Harville (1975), and Tallis (1985) indices are the same. Xie and Xu (1997) and Ceron-Rojas and Crossa (2018, Chapter 9) extended the DMLPSI and OMLPSI to the constrained context, respectively. The Xie and Xu (1997) index, however, is not an optimum constrained multistage index because their approach is based on the single-stage Tallis (1962) constrained index theory, which is not an optimum index (see Ceron-Rojas and Crossa, 2018, Chapter 3, for details).

Based on the Mallard (1972) constrained phenotypic single-stage index theory, which is an optimum singlestage constrained index (see Ceron-Rojas and Crossa, 2018, Chapter 3, for details), in this work, we extend the OMLPSI and DMLPSI to the constrained multistage selection context. We will denote the OMLPSI and DMLPSI as OCMLPSI (optimum constrained multistage LPSI) and DCMLPSI (decorrelated constrained multistage LPSI), respectively. The main difference between the OCMLPSI and the DCMLPSI is that the OCMLPSI imposes only one restriction when solving the OMLPSI equations to obtain its vector of coefficients, whereas the DCMLPSI imposes two restrictions. The OCMLPSI solves the OMLPSI equations subject to the restriction that the covariance between the OCMLPSI and some linear combinations of the genotypes involved be equal to a vector of predetermined proportional gains (or constraints) imposed by the breeder, whereas the DCMLPSI imposes the additional restriction that the covariance between DCMLPSI values at different stages be zero. This additional restriction negatively affects the DCMLPSI selection response and expected genetic gain values per trait after the first stage.

One of the purposes of conducting a multistage selection is to reduce the cost and still obtain a reasonable gain. This means that the OCMLPSI and DCMLPSI could also be optimized with respect to aggregated economic gain and cost associated with obtain measures on each trait, but in this work, that problem was not considered. In a two-stage context, Namkoong (1970) has detailed how this last problem could be solved for the OMLPSI, whereas Xu and Muir (1992) have described that problem in the DMLPSI context.

We compared the relative efficiency of OCMLPSI and DCMLPSI under the assumption that the net genetic merit and the OCMLPSI and DCMLPSI values have joint multivariate normal distribution. We corroborated the normality assumption at Stage 2 using graphical methods and normality tests (Shapiro and Wilk, 1965; Mardia, 1980). Under this assumption, the regression of the net genetic merit on any linear function of the phenotypic values is linear (Kempthorne and Nordskog, 1959) and the selection response and expected genetic gain per trait results for two or more stages can be summarized arithmetically (Cochran, 1951; Young, 1964). We used two criteria to compare the efficiency of both indices. The first criterion was that the total selection response of each index must be lower than or equal to the SCLPSI selection response (Young, 1964; Saxton, 1983; Ceron-Rojas et al., 2019). The second criterion was that the expected genetic gain per trait values should be similar to the predetermined gains or constraints imposed by the breeder. We used one real and one simulated dataset, each with four traits, to validate OCMLPSI efficiency vs. DCMLPSI efficiency. The results of both datasets indicated that the OCMLPSI is the most efficient index for predicting the net genetic merit, and its accuracy when predicting the selection response and estimating the expected genetic gain per trait was higher than the DCMLPSI accuracy when predicting the selection response and estimating the expected genetic gain per trait. Thus, breeders should use OCMLPSI when making a constrained phenotypic selection.

Results of this study are the first ones comparing (with real and simulated data) the relative efficiencies of the OCMLPSI vs. DCMLPSI using the total selection response and expected genetic gain pert trait as the main criteria to compare the efficiency of both indices.

## MATERIALS AND METHODS

### Methods Objectives of the Constrained Multistage Linear Phenotypic Selection Indices

Let *m*_j_ be the population mean of the *j*th trait before selection. One of the main OCMLPSI (DCMLPSI) objectives is to change *m*_j_ to *m*_j_ + *d*_j_, where *d*_j_ is the *j*th (*j* = 1, 2, …, *r*; *r* = the number of constrained traits) constrained trait or the *j*th predetermined proportional gain imposed by the breeder on the OCMLPSI (DCMLPSI) expected genetic gain per trait (Mallard, 1972; Cerón-Rojas and Crossa, 2018, Chapter 3). Additional OCMLPSI (DCMLPSI) objectives are (i) to maximize the selection response; (ii) to predict the net genetic merit (H = **w′g**, where w**^′^** = [w_1_ w _2_ … w_n_] and **g^′^** = [g_1_ g_2_ … g_*n*_are 1 × *n* vectors of economic weights and true unobservable breeding values, respectively); and (iii) to select individuals with the highest *H* values as parents of the next generation.

### The Part and Whole Phenotypic Index Selection Method

Let y′ = [γ_1_ γ_2_ … γ_*n*_] be a 1 × *n* vector of scores for *n* traits and assume that we can select only *n_i_* of them at Stage *i* (*i* = 1, 2, …, *N*; *N* = number of stages) such that after *N* stages, *n* = *n*_1_ + *n*_2_ + … + *n*_N_, where *n_i_*. < *N* < *n*. We can partition **y** into *N* subvectors as *y′* = [**x**_1_ **x**_2_ … **x**_N_], where *x′_i_*. = [γ_1_ γ_2_… γ_*ni*_] is the subvector of **y** at Stage *i* (*i* = 1, 2, …, *N*). This means that at this stage, the *i*th index is *I_i_* = β_i1γi1_ + β_i2γi2_… β_in_i_γin_i__ = β_i_′x_i_ where β′_i_ = [β_i1_ β_i2_ … β_in_i__] is the index vector of coefficients, whereas x_i_ was defined earlier. Let

$$B_{0}^{\prime }=\left[\begin{array}{cccc} \beta_{1}^{\prime } & 0 & \ldots & 0\\ \beta_{1}^{\prime } & \beta_{2}^{\prime } & \ldots & 0\\ \vdots & \vdots & \ddots & \vdots \\ \beta_{1}^{\prime } & \beta_{2}^{\prime } & \ldots & \beta_{N}^{\prime } \end{array}\right]$$

be a transforming matrix; then, for each stage, we can construct an index as

$$\left[\begin{array}{c} I_{1}\\ I_{2}\\ \vdots \\ I_{N} \end{array}\right]=\left[\begin{array}{cccc} \beta_{1}^{\prime } & 0 & \ldots & 0\\ \beta_{1}^{\prime } & \beta_{2}^{\prime } & \ldots & 0\\ \vdots & \vdots & \ddots & \vdots \\ \beta_{1}^{\prime } & \beta_{2}^{\prime } & \ldots & \beta_{N}^{\prime } \end{array}\right]\left[\begin{array}{c} x_{1}\\ x_{2}\\ \vdots \\ x_{N} \end{array}\right]$$

(Xu and Muir, 1992; Cerón-Rojas et al., 2019). This last result indicates that until Stage *N* - 1, each index is partial, but at Stage *N,* *I_N_* = β′_1_x_1_ + β′_2_x_2_ + … +β′_*N*_x_*N*_ is a whole index.

Young (1964) called the foregoing procedure the *part and whole index selection method*. Xu and Muir (1992) called that selection procedure selection index updating because as traits become available, each subsequent index contains all traits available up to that stage. This method is more efficient than the independent culling selection method because it uses the genetic correlation among traits and all available information at each stage to predict the net genetic merit (Saxton, 1983). In addition, the independent culling selection method cannot impose constraints on the expected genetic gain of each trait, as the constrained index does.

### Genotypic and Phenotypic Covariance Matrices

Let g′ = [g_1_ g_2_ … g_n_], **x′** = [𝓛_1_ 𝓛_2_ … 𝓛_n_i__], and y′ = [γ_1_ γ_2_ … γ_n_] be vectors, as defined in the above subsections. Thus, the genotypic covariance matrix of vectors **x***_i_* and **g** for *N* stages is

$$\left[\begin{array}{c} \mathrm{Cov}\left(x_{1},g\right)\\ \mathrm{Cov}\left(x_{2},g\right)\\ \vdots \\ \mathrm{Cov}\left(x_{N},g\right) \end{array}\right]=\left[\begin{array}{c} G_{1}\\ G_{2}\\ \vdots \\ G_{N} \end{array}\right]=G$$

whereas the phenotypic covariance matrix of vector **y** is Var(**y**) = {**P**_*ij*_} = **P**, where Cov(**x**_i_, **g**) = **G**_i_ is the *i*th submatrix of **G**, and **P***_ij_* = Cov(x*i*, x*_j_*) is the *ij*th (*i,j* = 1, 2,…, *N*) submatrix of **P**.

To obtain the OCMLPSI (DCMLPSI) parameters, we need the following matrices:

[1a] $$Q_{ii}=\left[\begin{array}{cccc} P_{11} & P_{12} & \ldots & P_{1i}\\ P_{21} & P_{22} & \ldots & P_{2i}\\ \vdots & \vdots & \ddots & \vdots \\ P_{i1} & P_{i2} & \ldots & P_{ii} \end{array}\right],\;A_{i}=\left[\begin{array}{c} G_{1}\\ G_{2}\\ \vdots \\ G_{i} \end{array}\right]$$

which are submatrices of **P** and **G**, respectively. In Appendix A (Eq. [A1] to [A3]), we describe a method to estimate **P** and **G**.

Now suppose that the number of traits selected up to Stage *i* - 1 is *n_i-1_* and that at Stage *i* we select *n_i_* traits, such that *n_i_* ≤ *n_i-1_* (or *n_i-1_* < *n_i_*). Then, according to the *part and whole index selection method*, at Stage *i*, we shall have *n-1*. + *n_i_* traits. This means that the phenotypic covariance matrix [**Q**_*(i-1)i*_] obtained with the *n_i-1_* traits selected at Stage *i* - 1 and the total *n_i-1_* traits will be of size *n_i-1_*(*n_i-1_* + *n_i_*) and can be written as

[1b] $$Q_{\left(i-1\right)i}=\left\{s_{jc}\right\}$$

where *s_jc_* is the *jc*th phenotypic covariance value for *j* = 1, 2, …, *n_i-1_* and *c* = 1, 2, … (*n_i-1_ + n_i_*). In addition, *n_i-1_* and (*n_i-1_* + *n_i_*) are the numbers of rows and columns of matrix **Q**_(*i-1)i*_’ respectively. Equation [1b] indicates that **Q**_(*i-1)i*_ is a nonsquare and nonsymmetric matrix. Matrix **Q**_(*i-1)i*_ is useful for imposing the restrictions that make the DCMLPSI values independent among stages (see Cerón-Rojas et al., 2019, for details).

### Selection Response at Stage i

At Stage *i*, the selection response (*R_i_*) is the *i*th net genetic merit (*H* = w′g) mean of the selected population and can be written as

[2] $$R_{i}=k_{i}\sigma_{H}\rho_{HI_{i}}$$

where *k_i_* is the selection intensity (Xu and Muir, 1992; Cerón Rojas et al., 2019), $\sigma_{H}=\sqrt{w\prime \mathrm{Cw}}$ is the standard deviation of *H* = **w′g**, Var(**g**) = **C** is the covariance matrix of **g**, and ρ_HI_i__ is the correlation between *H* = **w′g** and the index at Stage *i* (***I**_i_* = β′_i_ x_i_). For *N* stages, the total selection response is *R*_*t*_ = *R*_1_ + *R*_2_ + … + *R*_*N*_ (Cochran, 1951; Young, 1964). Equation [2] indicates that the genetic gain that can be achieved in R_*i*_ by selecting for several traits simultaneously within a population of animals or plants is the product of *k_i_*, <_H_, and ρ_*HI*_ (Kempthorne and Nordskog, 1959). Selection intensity is limited by the rate of reproduction of each species, whereas <_H_ is beyond human control; hence, the greatest opportunity for increasing selection progress is by ensuring that ρ*_HI_i__* is as large as possible (Hazel 1943). Equation [2] is a useful criterion for comparing the efficiency of different types of indices to predict the net genetic merit (*H* = **w′g**; e.g., OCMLPSI efficiency vs. DCMLPSI efficiency). We would expect that the greater Eq. [2] is, the more effective OCMLPSI (DCMLPSI) is at predicting *H* = w ‘g. In the multistage selection index context, however, one main restriction is that the whole OCMLPSI (DCMLPSI) selection response be lower than or equal to the SCLPSI response (Saxton, 1983; Cerón-Rojas et al., 2019).

### Expected Genetic Gain per Trait at Stage i

The expected genetic gain per trait at Stage *i* (**E**_*i*_, or multitrait selection response) is the covariance between the true breeding value vector (**g**) and the *I_i_* = β′_*i*_x_*i*_ value weighted by its standard deviation $\left(\sigma_{1_{i}=}\sqrt{{\beta \prime }_{i}Q_{\mathrm{ii}}\;\beta_{i}}\right)$ and multiplied by the selection intensity (*k_i_*), so that

[3] $$E_{i}=k_{i}\frac{A_{i}^{\prime }\beta_{i}}{\sigma_{I_{i}}}$$

We defined all the parameters of Eq. [3] previously. In the univariate and single-stage breeding scheme, Eq. [3] is the same as the selection response. For *N* stages, the total expected genetic gain per trait is **E**_*t*_ = **E**_*1*_ + **E***_2_* + … + **E**_*n*_ (Cochran, 1951; Young, 1964).

In the OCMLPSI context, we will minimize the mean squared difference between the net genetic merit H = w ‘g and the index *I_i_* = β′_i_x_i_ {i.e., E(H-I_i_)^2^]} with respect to the vector of coefficients β_i_(i = 1,2, …, N) under the assumption that Eq. [3] values are equal to the *d_j_* (j = 1, 2, …, r; r = number of constraints) values imposed by the breeder. The resulting vector of coefficients (β_i_) should maximize Eq. [2] and make the Eq. [3] values be near the d_*j*_ value. In the DCMLPSI context, however, it is necessary to impose the additional restriction that the DCMLPSI values among stages are independent, as we shall see in the next two subsections.

### The OCMLPSI Vector of Coefficients at Stage i

Let d’ = [d_1_ d_2_ … d_r_] be a vector 1 X r of constraints or predetermined proportional gains per trait imposed by the breeder, and

$$D^{\prime }=\;\left[\begin{array}{ccccc} d_{r} & 0 & \ldots & 0 & -d_{1}\\ 0 & d_{r} & \ldots & 0 & -d_{2}\\ \vdots & \vdots & \ddots & \vdots & \vdots \\ 0 & 0 & \ldots & d_{r} & -d_{r-1} \end{array}\right]$$

be a Mallard (1972) matrix of size (r — 1)r, where d_*j*_ (*j* = 1, 2, …, r) is the jth element of vector d’. In addition, let U’ be a Kempthorne and Nordskog (1959) matrix (n — r)n (n = number of traits and r = number of constraints) of 1s and 0s, where 1 indicates that the trait is constrained and 0 indicates that the trait has no constraints (see Cerón-Rojas and Crossa, 2018, Chapter 3, for details). According to the single-stage Mallard (1972) constrained index theory, to obtain the OCMLPSI vector of coefficients at Stage *i*, we need to minimize the mean squared difference between the net genetic merit *H* = **w′g** and the index *I_i_*= β′_i_x_i_ {i.e., i.e., E(H - I^i^)^2^]} under the restrictions **M**′ β_*I*_ = 0, where **M**’_*i*_ = **D** ‘**U**’**A**’_*i*_. and **A**’_*i*_ was defined in Eq. [1a].

Suppose that matrices **Q**_*ii*_’ **U**, and **A**’_*i*_’ and vectors **d** and **w** are known at Stage *i*; then, it is necessary to minimize the function

[4] $$f_{○}\left(\beta_{i},u\right)=\beta_{i}^{\prime }Q_{ii}\beta_{i}-2{\mathrm{wA}}_{i}^{\prime }\beta_{i}+2u^{\prime }M_{i}^{\prime }\beta_{i}$$

with respect to the vector of coefficients β_*i*_ and the vector of Lagrange multipliers **u**’ = [*u_1_ u_2_ … u_r-1_*] The OCMLPSI vector of coefficients at Stage *i* is

[5] $$\beta_{i}=K_{○i}\delta_{i}$$

where δ*_i_* = **Q**_ii_^-1^ is the inverse of matrix **Q**_*ii*_’ and **A**_*i*_ and **w** were defined earlier. In addition, **K**_Oi_ = [**I**_i_ - **F**_Oi_ = **Q**_ii_^-1^**M**)^-1^**M**^′^_i_, and **I**_i_. is an identity matrix of the same size as **Q**_ii_. When **D** = **U**, the vector of coefficients of Eq. [5] imposes null restrictions, and when **D** = **U** and **U** is a null matrix, Eq. [5] is equal to δ_i_ = **Q**_ii__-1_**A**_i_**w**, the vector of coefficients of the OMLPSI (Cerón-Rojas et al., 2019). Thus, the OCMLPSI is more general and includes the multistage null phenotypic restricted index (Kempthorne and Nordskog, 1959; Xie and Xu, 1997) and the OMLPSI as particular cases (Cerón- Rojas and Crossa, 2018, Chapter 9).

### The DCMLPSI Vector of Coefficients at Stage i

Let I_Di-1_ = **b**^′^_i-1_**x**_i-1_ and I_*Di*_ = **b**^’^_i_**x**_i_ be the DCMLPSIs at Stages *i* - 1 and *i*, respectively. We shall obtain the DCMLPSI vector of coefficients at Stage *i* with the additional restriction that the covariance between the DCMLPSI values until Stage *i* _ 1 with the I_Di_ = **b**_i_^’^**x**_i_ values be null. Let **J**^′^_i - 1_ =[I^D1^ I_D2_ … I_D(i-1)_] be a vector of DCMLPSIs values until Stage *i* — 1 such that the covariance between I_Di_ and **J**_i i - 1_ will be null. Xu and Muir (1992) and Xie and Xu (1997) showed that the covariance between **I**_Di_ and **J**_i - 1_ is null when **B**′_D(i-1)_ **Q**_(i-1)i_**b**_i_ = 0, where

$$B_{D\left(i-1\right)}^{\prime }=\left[\begin{array}{cccc} b_{1}^{\prime } & 0 & \ldots & 0\\ b_{1}^{\prime } & b_{2}^{\prime } & \ldots & 0\\ \vdots & \vdots & \ddots & \vdots \\ b_{1}^{\prime } & b_{2}^{\prime } & \ldots & b_{i-1}^{\prime } \end{array}\right]\prime$$

**b**^′^_i - 1_ = [*b*_(*i* -1)1_ *b*_(**i** - 1)2_ … **b**_(*i*-1)n_(*i*-1)__] is the DCMLPSI vector of coefficients at Stage *i* - 1, Q(_*i* - 1)i_ was defined in Eq. [1b] and **b***_i_* is the DCMLPSI vector of coefficients at Stage *i*. Thus, to obtain **b***_i_*, we need to minimize the mean squared difference between H = **w**^′^**g** I_Di_ = **b**^′^*_i_***x**_i_ {i.e., E[(H - I_Di_)^2^]}, under the joint restrictions **M**^′^_i_**b**_i_ = 0 Cov(I_Di_,**J**_i - 1_)=**B**′_D(i - 1) = **Q**__D(i - 1)_ **b**_i_ = 0.

Let **S**_*i*(*i* - 1)_ = **Q**_*i*(*i*-1)_ **B**_*D*(*i*-1)_ be the transpose of matrix **S**_*i*(*i-1*)_ and assume that matrices **Q**_ii_, **Q**_*i*(*i*-1)_, **U**, and **A**_i_ and **A***i*. and vectors **d** and **w** are known. To minimize *E*[(*H - I_Di_*)^2^] under the restrictions M′*_i_*b*_i_* = 0 and **S**_(*i* - 1)*i*_ = **B**^′^_*D*(*i*-1)_**Q**_(*i*-1)_*i***b**_*i*_ = 0, it is necessary to minimize the function

[6] $$f_{D}\left(b_{i},u,v\right)=b_{i}^{\prime }Q_{ii}b_{i}-2wA_{i}^{\prime }b_{i}+2u^{\prime }M_{i}^{\prime }b_{i}+2v^{\prime }S_{\left(i-1\right)i}b_{i}$$

with respect to the vector of coefficients **b***_i_* and the vector of Lagrange multipliers **u**^′^ = [*u*_1_ **u**_2_ *u*_*r* - 1_] and *v*^′^ = [*v*_1_ *v*_2_ … *v*_*i* - 1_]. The only difference between Eq. [4] and Eq. [6] is the term 2**v**^′^**S**_(*i* - 1)_*i***b***i*. The DCMLPSI vector of coefficients at Stage *i* is

[7] $$b_{i}=K_{Di}\delta_{i}$$

where **δ**_i_ = **Q**_ii_^-1^ **A**_i_ **w**, **Q**_ii_^-1^ **A**_i_ and **w** were defined in Eq. [5]. In addition, **K**_*Di*_ and [**I**_*i*_ - **F**_*Di*_], **F**_*Di*_ = **Q**_*ii*_^-1^**V**_i_(**V**′_i_**Q**_11_^-1^**V**_*i*_)^-1^, **V**_i_ = [**M**_*i*_ **S**_*i*(*i* - 1)_], **V**′_i_ = [**M**′_i_ **S**_(*i* - 1)*i*_], and **I***_i_* is an identity matrix of the same size as Q_*ii*_. Thus, the only difference between Eq. [5] and [7] is matrix **S**_*i*(*i* - 1)_. When matrix **S**_*i*(*i* - 1)_ is null, Eq. [5] and [7] are the same, as we would expect. If **D** = **U**, Eq. [6] and [7] impose null restrictions; in such cases, we shall have a multistage index similar to the Kempthorne and Nordskog (1959) index.

According to Eq. [5] and [7] results, matrices **K**_o*i*_ and **K**_*Di*_ transform the OMLPSI vector of coefficients (**δ**_i_ = **Q**_ii_^-1^**A**_*i*_**w**) into the OCMLPSI and the DCMLPSI vectors of coefficients, respectively.

### Maximized Selection Response at Stage i

It can be shown (Cerón-Rojas and Crossa, 2018, Chapter 9) that when we use Eq. [5] and [7] in Eq. [2], we obtain

[8a] $$R_{Oi}=k_{Oi}\sqrt{\beta_{i}^{\prime }Q_{ii}\beta_{i}}$$

and

[8b] $$R_{Di}=k_{Di}\sqrt{b_{i}^{\prime }Q_{ii}b_{i}}$$

which are the maximized OCMLPSI and DCMLPSI selection responses at Stage i, respectively. Although in Eq. [2] the selection response can take any value, in Eq. [8a] and [8b], *R*_O*i*_ and *R*_*Di*_ give the maximum value of Eq. [2] for the OCMLPSI and DCMLPSI, respectively. In addition, in practice k_*Oi*_ and k_*Di*_ are obtained with a different method; therefore, their values are generally different (i.e., k_*Oi*_ ≠ k_*Di*_). In this work, we obtained the k_*Oi*_ value in a two-stage breeding scheme according to Eq. [A6] (Appendix B), whereas we obtained the k_*Di*_ values according to the Xu and Muir (1992) method. We described a method to estimated Eq. [8a] and [8b] in Appendix B (Eq. [A1] to [A4b]).

The maximized total OCMLPSI and DCMLPSI selection responses for N stages can be written as as *R*_t_O__ = *R*_O1_ + *R*_O2_ + … + *R*_ON_ and *R*_t_D__ = *R*_D1_ + *R*_D2_ + … + *R*_DN_, respectively.

### Maximized Expected Genetic Gain per Trait at Stage i

Using Eq. [5] and [7] in Eq. [3], the OCMLPSI and DMLPSI expected genetic gains per trait at Stage *i* can be written as

[9a] $$E_{Oi}=k_{Oi}\frac{A_{i}^{\prime }\beta_{i}}{\sqrt{\beta_{i}^{\prime }Q_{ii}\beta_{i}}}$$

and

[9b] $$E_{Di}=k_{Di}\frac{A_{i}^{\prime }b_{i}}{\sqrt{b_{i}^{\prime }Q_{ii}b_{i}}}$$

respectively. We defined all the parameters of Eq. [9a] and [9b] earlier. The maximized total OCMLPSI and DMLPSI expected genetic gains per trait for N stages can be written as **E**_t_*0*__ = E_O1_ + E_o2_ + … + E_*ON*_ and E_*tD*_ = E_*D1*_ + E_*D2*_ + … + E_*DN*_’ respectively. We described a method to estimated Eq. [9a] and [9b] in Appendix B (Eq. [A1] to [A3], and Eq. [A5a] and [A5b]).

### Efficiency when Predicting the Net Genetic Merit

According to Lande and Thompson (1990) and Moreau et al. (1998), the efficiency of the indices when predicting the net genetic merit, in percentage terms, is

[10] $$\emptyset =100\left(T-1\right)$$

where *T* = *R*_Oi_/*R*_Di_, *R*_Oi_ denotes the OCMLPSI selection response and R_*Di*_ the DCMLPSI selection response. Therefore, when Ø is null, the efficiency of both indices is the same; when Ø> 0, the efficiency ofthe OCMLPSI is higher than that of the DCMLPSI, and when Ø < 0, DCMLPSI efficiency is higher than OCMLPSI efficiency for predicting the net genetic merit.

An additional criterion for comparing the indices’ efficiency is that the total selection response *R*_*t*_ = *R*_1_ + *R*_2_ of each index should be lower than or equal to the single-stage constrained index selection response (*R* = *kσ_I_*), i.e., R_*t*_ ≤ R (see Cerón-Rojas et al., 2019, for details).

### Adjusting the OCMLPSI Covariance Matrices at Stage 2

At Stage 2, the phenotypic covariance matrix is

$$P=\left[\begin{array}{cc} P_{1} & P_{12}\\ P_{21} & P_{2} \end{array}\right]=Q_{22}$$

whereas the genotypic covariance matrix is

(Eq. [1a]) $$G=\left[\begin{array}{c} G_{1}\\ G_{2} \end{array}\right]=A_{2}$$

These matrices are affected by prior selection on I_1_ = β′x_1_. It is thus necessary to adjust them to take into consideration the I_1_ = β’_1_x_1_ effects on them. According to Cochran (1951) and Cunningham (1975), both matrices can be adjusted as follows:

[11a] $$P^{*}=P-a\frac{\left[\begin{array}{c} P_{1}\\ P_{21} \end{array}\right]\beta_{1}\beta_{1}^{\prime }\left[\begin{array}{cc} P_{1} & P_{21} \end{array}\right]}{\beta_{1}^{\prime }P_{1}\beta_{1}}$$

and

[11b] $$G^{*}=G-a\frac{G_{1}^{\prime }\beta_{1}\beta_{1}^{\prime }G_{1}}{\beta_{1}^{\prime }P_{1}\beta_{1}}$$

where P* and G* are the adjusted matrix, a = *k*_O1_(*k*_O1_ - *u*), *k_o1_* is the selection intensity at Stage 1, *u* is the truncation point when *I*_1_ = β’_1_x_1_ is applied, **P**_1_ = Var(x_1_) and G_1_ = Cov(**x**_1_’ **g**). Thus, the maximized OCMLPSI selection response (Eq. [8a]) and expected genetic gains (Eq. [9a]) at Stage 2 can be written as $R_{O2\;}=\kappa_{O2}\sqrt{\beta \prime_{2}P*\beta_{2}}\text{and}E_{O2\;}=\kappa_{O2}\left(G*\beta_{2}/\sqrt{\beta \prime_{2}P*\beta_{2}}\right)$, respectively.

### Test of the OCMLPSI (DCMLPSI) Normality Assumption

Several authors (Shapiro and Wilk 1965; Mardia 1980; Mohd- Razali and Bee-Wah 2011; Rani Das and Rahmatullah Imon, 2016) have given details of how to perform a normality test procedure on a dataset and many statistical packages provide graphs and normality tests.

We corroborated the OCMLPSI (DCMLPSI) normality assumption at Stage 2 with a simulated dataset using a graphical method (histograms) and analytical test procedures (the Shapiro—Wilk and Kolmogorov—Smirnov normality test). The corroboration procedure was as follows. In a two-stage context, let p = q_1_q_2_ be the fixed total proportion retained, where q_1_ and q_2_ denote the proportion selected at Stage 1 and 2, respectively, and let n be the size of the simulated dataset at Stage 1; then, nq_1_ will be the size of the selected individuals at Stage 1. We used the information of nq_1_ individuals at Stage to construct graphs and statistical tests to corroborate the OCMLPSI (DCMLPSI) normality assumption.

## Materials

### Real Dataset

The number of genotypes in this real data set was 3330 and the vector of economic weights (**w**) was **w**′ = [19.54 _3.56 17.01 _2.51]. This dataset comes from a commercial egg poultry line (Akbar et al., 1984) and we used it to illustrate the indices’ theoretical results obtained in this work. The estimated phenotypic (P) and genotypic (**Ĉ**) covariance matrices among the rate of lay (RL, number of eggs), age at sexual maturity (SM, d), egg weight (EW, kg), and body weight (BW, kg) were

$$\hat{P}=\left[\begin{array}{cccc} 240.57 & -95.62 & 2.06 & 54.40\\ -95.62 & 167.20 & 4.58 & 15.36\\ 2.06 & 4.58 & 22.80 & 37.20\\ 54.40 & 15.36 & 37.20 & 516.11 \end{array}\right]$$

and

$$\hat{C}=\left[\begin{array}{cccc} 29.86 & -17.90 & -4.13 & -1.75\\ -17.90 & 18.56 & 1.49 & -4.88\\ -4.13 & 1.49 & 9.24 & 16.66\\ -1.75 & -4.88 & 16.66 & 179.73 \end{array}\right]$$

The total proportions (*p*) of retained values for this dataset were *p* = 0.05, 0.10, 0.20, and 0.30 for both indices.

For illustration purposes only, at Stage 1, we selected RL, SM, and EW, where RL and SM were constrained by the vector of predetermined restrictions **d**’ = [3 -1] and matrices

$$U^{\prime }=\left[\begin{array}{cccc} 1 & 0 & 0 & 0\\ 0 & 1 & 0 & 0 \end{array}\right]$$

and **D**’ = [-1 -3] for both stages, whereas at Stage 2, we selected trait BW only. The vectors of records at Stages 1 and 2 were **x**’_1_ = [RL SM EW] and **y**’ = [**x**’_1_ **x**’_2_], respectively, where **x**_2_ = BW. At Stage 1, the estimated phenotypic (**Ô**_11_) and genotypic (**Â**_1_) covariance matrices were

$${\hat{Q}}_{11}=\left[\begin{array}{ccc} 240.57 & -95.62 & 2.06\\ -95.62 & 167.20 & 4.58\\ 2.06 & 4.58 & 22.80 \end{array}\right]$$

and

$${\hat{A}}_{1}=\left[\begin{array}{cccc} 29.86 & -17.90 & -4.13 & -1.75\\ -17.90 & 18.56 & 1.49 & -4.88\\ -4.13 & 1.49 & 9.24 & 16.66 \end{array}\right]$$

respectively, whereas the estimated covariance matrix of the traits at Stage 1 with traits at Stage 2 (**Ô**_12_) was

$${\hat{Q}}_{12}=\left[\begin{array}{ccc} 240.57 & -95.62 & 2.06\\ -95.62 & 167.20 & 4.58\\ 2.06 & 4.58 & 22.80 \end{array}\begin{array}{c} 54.40\\ 15.36\\ 37.20 \end{array}\right]$$

### Simulated Datasets

These datasets are available in the *Application of a Genomics Selection Index to Real and Simulated Data* repository, at http://hdl.handle.net/11529/10199. They were simulated by Ceron-Rojas et al. (2015) with QU-GENE software (Podlich and Cooper, 1998) using 2500 molecular markers and 315 quantitative trait loci (QTLs) for eight phenotypic selection cycles (C0—C7), each with four traits (T_1_’ T_2_’ T_3_’ and T_4_)’ 500 genotypes and four replicates for each genotype. The authors distributed the markers uniformly across 10 chromosomes and the QTLs randomly across the 10 chromosomes to simulate maize (*Zea mays L.*) populations. A different number of QTLs affected each of the four traits: 300, 100, 60, and 40, respectively. The common QTLs affecting the traits generated genotypic correlations of -0.5, 0.4, 0.3, -0.3, —0.2, and 0.1 between T_1_ and T_2_’ T_1_’ and T_3_’ T_1_ and T_4_’ T_2_’ and T_3_’ T_2_ and T_4_’ T_3_ and T_4_’ respectively. The economic weights for T_1_’ T_2_’ T_3_’ and T_4_ were 1, —1, 1, and 1, respectively.

We used four phenotypic selection cycles (C1—C4) with *p* = 0.01, 0.10, and 0.30 in each cycle. At Stage 1 we selected *T_1_’* T_2_’ and T_3_’ where T_1_ and T_2_ were constrained with vector **d**’ = [5 —2] and matrices

$$U^{\prime }=\left[\begin{array}{cccc} 1 & 0 & 0 & 0\\ 0 & 1 & 0 & 0 \end{array}\right]$$

and **D**’ = [—2 —5] for both stages. At Stage 2 we selected only trait T_4_; thus, the vector of observations at Stage 1 was **x**’ _1_ = [T_1_ T_2_ T_3_] and at Stage 2,**y** ’ = [**x**’_1_ **x**‘_2_]’ where x_2_ = T_4_.

## RESULTS Real Data

### Truncation Points, Proportion Retained, and Selection Intensities for Two Stages

**Fig. 1:**
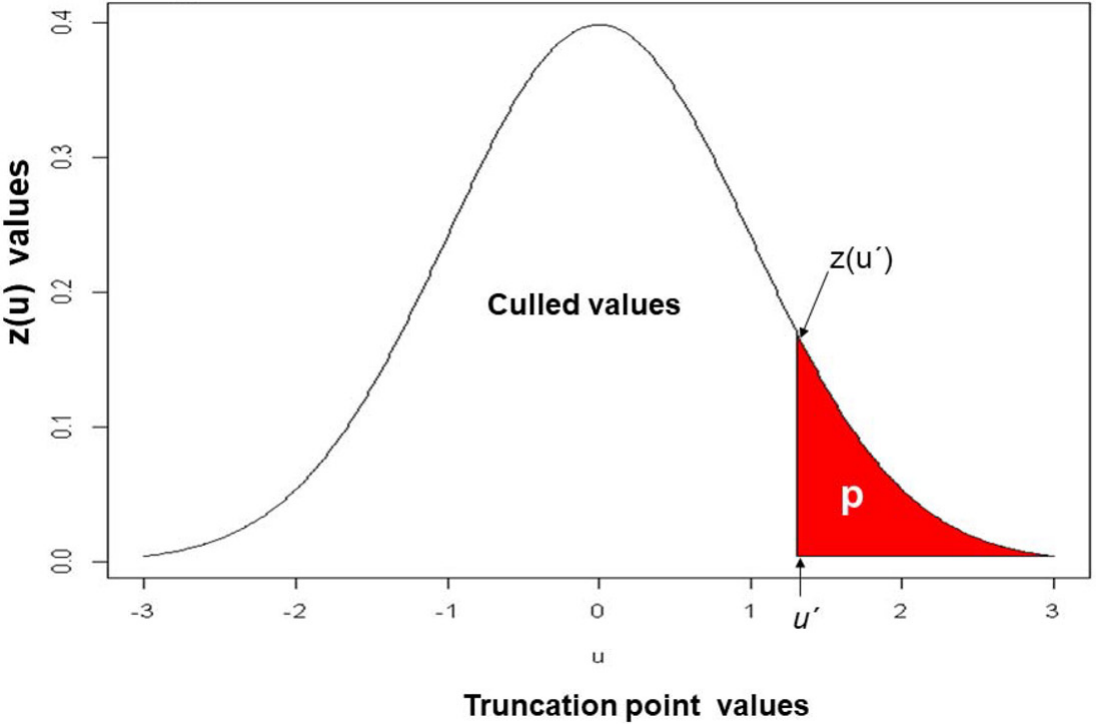
Theoretical relationship between the truncation points (u), the proportion retained (p), and the density values [z(u)] of the truncation points (after Ceron-Rojas et al., 2019).

**Fig. 2:**
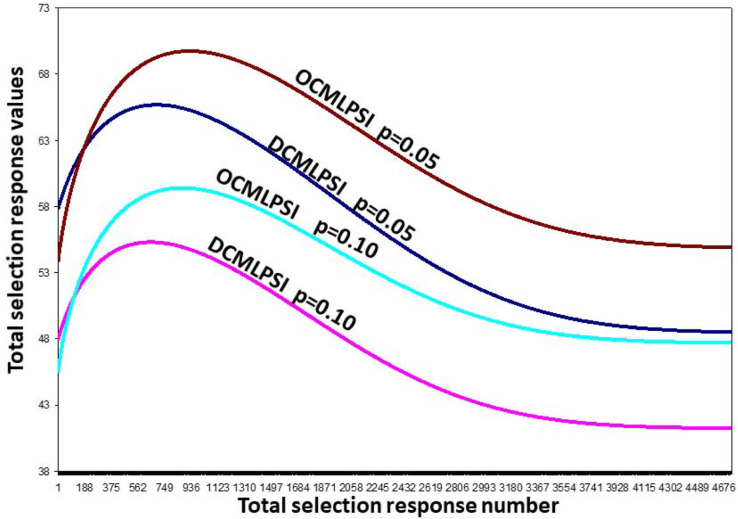
Distribution of the total estimated optimum and decorrelated constrained multistage linear phenotypic selection index (OCMLPSI and DCMLPSI, respectively) selection response values for a real dataset, and the fixed total proportion retained (p) = 0.05 and 0.10.

Figure 1 shows the relationship among the truncation points (*u*_1_ and *u*_2_), the total proportion retained (*p* = q_1_q_2_) and the heights of the ordinate of the normal curve: *z*(*u*_1_) = *e*^-0.5^*u*_1_^2^ and *z*(*u*_2_) We found the OCMLPSI selection intensity for Stages 1 [*k*_1_ = *z*(*u*_1_)/*q*_2_] according to Eq. [A6] (Appendix B) as follows. For a fixed value of *p* = *q*_1_*q*_2_ (e.g., *p* = 0.05), we used an iterative process with an R code. By successively changing the possible values of *q*_1_ (*q*_2_ = *p*/ *q*_1_), *u*_1_, and *u*_2_, we found the maximum value of the estimated total OCMLPSI (DCMLPSI) selection response,R^_t_ = R^_1_ + R^_2_ (Fig. 2). For example, for the real dataset and *p* = 0.05, the estimated total OCMLPSI selection response was R^_t_ = R^_1_ + R^_2_ = 25.592*k*_1_ + 26.580*k*_2_ = 69.75, where _1_ =*k*_1_*ô*_I__i_ = 33.265 and R^_2_ = *k*_2_ = 36.481 were the estimated selection responses at each stage (Table 1), whereas *ô*_1__2_ = 26.579 **Î**_1_ and **Î**_2_ were the estimated standard deviations of the variance of *Î*_1_ and *Î*_2_ for Stages 1 and 2, respectively. Thus, for this dataset, the values of the truncation points (*u*_1_ = 0.710 and *u*_2_ = 0.81), proportions retained (*q*_1_ = 0.24 and *q*_2_ = 0.21) and selection intensity (*k*_1_ = 1.30 and k_2_ = 1.37), at both stages, were those associated with the maximum estimated total OCMLPSI selection response **Ȓ***_t_*= 69.75 value. Table 1, presents additional truncation points, proportions retained, selection intensities for *p* = q_1_q_2_ = 0.10, 0.20, and 0.30, associated to the OCMLPSI^1^ a^2^nd DCMLPSI selection responses.

**Table 1.** Real data for total proportion (*p*) retained, estimated optimum and decorrelated constrained multistage linear phenotypic selection index (OCMLPSI and DCMLPSI, respectively) truncationpoints (*u*_1_ and *u*_2_), proportions retained (*q*_1_ selection intensities (*k*_1_ and *k*_2_), and selection response (*Ȓ*_1_, *Ȓ*_2_, and *Ȓ*_t_ =*Ȓ*_1_ + *Ȓ*_2_) for Stages 1 and 2. Values of >R >correspond to the one-stage estimated constrained linear phenotypic selection index selection response.

Index	*P*	*u*_i_	*U*_2_	*q*_1_	*q*_2_	*k*_i_	*k*_2_	*Ȓ*_1_	*Ȓ*_2_	*Ȓ*_t_	*Ȓ*
OCMLPSI	0.05	0.71	0.81	0.24	0.21	1.30	1.37	33.26	36.48	69.75	71.66
	0.10	0.41	0.55	0.34	0.29	1.07	1.18	27.45	31.95	59.40	60.96
	0.20	0.03	0.23	0.49	0.41	0.81	0.95	20.85	26.65	47.50	48.63
	0.30	−0.25	0.00	0.60	0.50	0.64	0.80	16.48	22.96	39.44	40.26
DCMLPSI	0.05	0.86	0.65	0.19	0.26	1.42	1.25	36.29	29.40	65.69	71.66
	0.10	0.58	0.37	0.28	0.35	1.20	1.05	30.65	24.66	55.30	60.96
	0.20	0.23	0.03	0.41	0.49	0.95	0.82	24.23	19.24	43.47	48.63
	0.30	−0.03	−0.22	0.51	0.59	0.78	0.67	19.91	15.64	35.55	40.26

### Estimated OCMLPSI Selection Response for Two Stages

In the one-stage case, the selection intensity for *p* = 0.05 was *k* = 2.063, and the SCLPSI selection response was Ȓ = 71.66 (see Cerón-Rojas and Crossa, 2018, Chapter 3, for details). According to the results detailed in the paragraph above and to Young (1^1964^) and Saxton (1983), the maximum estimated total OCMLPSI selection response (*Ȓ*_t_= 69.75) value should be lower than or equal to the estimated SCLPSI response (*Ȓ* = 71.66). For this dataset, *Ȓ*_t_= 69.75 explained 97.33% of the *Ȓ* = 71.66 value. That is, *Ȓ*_t_ = 69.75 and R = 71.66 were very similar.

In Table 1, we present additional maximum estimated OCMLPSI selection response values for *p* = *q*_1_*q*_2_ = 0.10, 0.20, and 0.30. For each of the latter three p values, the maximum estimated total OCMLPSI selection responses explained 97.44, 97.68, and 97.96%, respectively, of the SCLPSI selection response values. Thus, for this real dataset, the estimated total OCMLPSI selection response and the estimated SCLPSI selection response were very similar, as we would expect.

### Estimated DCMLPSI Selection Response for Two Stages

For both stages, the estimated DCMLPSI vectors of coefficients (Eq. [7]) were **b**’_1_ = [1.809 1.132 1.065] and **b**’_2_ = [0.349 —0.262 1.743 —1.076]. Because at Stage 1 the restriction matrix **S**(_*i*-1)*i*_ was null [**S**_(*i*-1)*i*_=0], the estimated DCMLPSI vector of coefficients was the same as the OCMLPSI vector of coefficients (i.e.,**b^**_1_ = β^′_1_; Eq. [5] and [7]); however, at Stage 2, **S**(**i** - 1)**i** ≠ and β^′_2_.

For *p* = 0.05, the selection intensities obtained with the Xu and Muir (1992, Eq. [19]) method were *k*_1_ = 1.42 and *k*_2_ = 1.25 at Stages 1 and 2, respectively, from where the estimated maximized selection responses for both stages were *Ȓ*_1_ = 39.29 and *Ȓ*_2_ = 29.40 (Appendix A, Eq. [A4b]), whereas R^_t_ = R^_1_R^_2_ = 65.69 was the total estimated selection response. This means that *Ȓ*_t_ = 65.69 explained 91.67% of the estimated SCLPSI selection response (*Ȓ* = 71.66) value.

Table 1 presents additional maximum estimated DCMLPSI selection response values when *p* = *q*_1_*q*_2_ = 0.10, 0.20, and 0.30. For 0.10, 0.20 and 0.30, the estimated total selection response explained 90.72, 89.40, and 88.30%, respectively, of the estimated SCLPSI selection response.

The results of the last two subsections indicate that the average of the estimated total DCMLPSI and OCMLPSI selection responses explained 90 and 97.60%, respectively, of the average estimated SCLPSI selection response for all *p* values. This means that the average of the estimated total OCMLPSI selection response was 7.60% closer to the estimated SCLPSI selection response than the average of the estimated total DCMLPSI selection response. We explain the loss of DCMLPSI efficiency by noting that when DCMLPSI obtained its vector of coefficients, it incorporated an additional restriction, which made the DCMLPSI values independent at different stages. Xu and Muir (1992) and Xie and Xu (1997) indicated that the loss of efficiency is justified because their method for obtaining the selection intensities and total responses gives the breeder the opportunity to implement an unlimited number of selection stages, which otherwise would be very difficult or impossible to do.

### Estimated OCMLPSI Expected Genetic Gain per Trait for Two Stages

Let *p* = 0.05 (*k*_1_ = 1.30 and *k*_2_ = 1.37); then, the estimated OCMLPSI expected genetic gains per trait (Appendix A, Eq. [A5a]) for both stages were E^^′^_1_ = [1.49 -0.50 0.21 0.46] and E^^′^_2_ = [1.92 -0.64 0.01 -8.02], while E^^′^ = E^′_1_ + E^′_2_ = [3.41 .1.14 0.21 .7.56] [3.41 -1.14 0.21 -7.56] was the total estimated expected genetic gain per trait. Each E^′_t_ value was associated with the mean of traits rate of lay (RL, number of eggs), age at sexual maturity (SM, days), egg weight (EW, kg) and body weight (BW, kg). We constrained traits RL and SM by vector *d*′ =[3 -1] values. This means that the E^′_t_ values associated with traits RL and SM (3.41 and -1.14, respectively) overestimated the **d**’ = [3 —1] values by 10.33 and 14%, respectively.

Table 2 presents additional estimated expected genetic gains per traits RL, SM, EW, and BW for both stages and *p* = 0.10, 0.20, and 0.30. For these last three *p* values, the estimated total OCMLPSI expected genetic gains per traits RL and SM explained 95, 74, and 59% of each **d**’ = [3 -1] value, respectively. That is, the accuracy of the estimated OCMLPSI expected genetic gains per trait decreased when the p values increased from 0.10 to 0.30. For this real dataset, the optimum expected genetic gain per trait efficiency occurred when *p* = 0.10.

**Table 2.** Total proportion (p) retained; estimated optimum and decorrelated constrained multistage linear phenotypic selection index (OCMLPSI and DCMLPSI, respectively) expected genetic gains per trait for four real traits: rate of lay (RL), age at sexual maturity (SM), egg weight (EW), and body weight (BW). Traits RL and SM were constrained with vector d’ (a vector of constraints or predetermined proportional gains per trait imposed by the breeder) = [3 -1] values.

Index	Stage 1						Stage 2		Total			
	*p*	RL	SM	EW	BW	RL	SM	EW	BW	RL	SM	EW	BW
no. eggs	d		kg		no. eggs	d		kg		no. eggs	d		kg	
OCMLPSI	0.05	1.49	−0.50	0.21	0.46	1.92	−0.64	0.01	−8.02	3.41	−1.14	0.21	−7.56
0.10	1.23	−0.41	0.17	0.38	1.62	−0.54	0.01	−6.72	2.85	−0.95	0.18	−6.34
0.20	0.93	−0.31	0.13	0.29	1.27	−0.42	0.01	−5.26	2.21	−0.74	0.14	−4.97
0.30	0.74	−0.25	0.10	0.23	1.04	−0.35	0.01	−4.30	1.78	−0.59	0.11	−4.07
DCMLPSI	0.05	1.63	−0.54	0.22	0.50	0.52	−0.17	0.19	−8.72	2.15	−0.72	0.03	−8.22
	0.10	1.37	−0.46	0.19	0.42	0.44	−0.15	0.16	−7.32	1.81	−0.60	0.03	−6.89
	0.20	1.09	−0.36	0.15	0.34	0.34	−0.11	0.13	−5.71	1.43	−0.48	0.02	−5.37
0.30	0.89	−0.30	0.12	0.28	0.28	−0.09 -	−0.10	−4.64	1.17	−0.39	0.02	−4.36

### Estimated DCMLPSI Expected Genetic Gain per Trait for Two Stages

For *p* = 0.05 (*k*_1_ = 1.42 and *k*_2_ = 1.25), the estimated DCMLPSI expected genetic gains per trait for both stages were E^′_1_ = [1.63 _0.54 0.22 0.50] and E^′_2_ = [0.52 _0.17 -0.19 -8.72], whereas E^′_t_ =E^′_1_ + E^′_2_ = [2.15 -0.72 0.03 -8.22] was the total estimated expected genetic gain per trait. Each E^′_t_ value is associated with traits RL (rate of lay, number of eggs), SM (age at sexual maturity, days), EW (egg weight, kg), and BW (body weight, kg), and traits RL and SM were constrained by vector **d**’ = [3 -1] values. The E^′_t_ values associated with RL and SM (2.15 and -0.72, respectively) explained only 71.67 and 72% of each **d**’ = [3 -1] value, respectively.

Table 2 presents additional estimated expected genetic gains per traits RL, SM, EW, and BW for both stages and *p* = 0.10, 0.20, and 0.30. For 0.10, 0.20, and 0.30, the estimated total expected genetic gains per traits RL and SM explained 60.33, 47.67, and 39% of each **d**′ = [3 -1] value, respectively. Thus, for this dataset, the estimated expected genetic gains per trait underestimated the **d**′ = [3 -1] values. We explained the loss of DCMLPSI accuracy, noting that when the DCMLPSI obtained its vector of coefficients, it incorporated an additional restriction to make the DCMLPSI values independent among stages. The average estimated DCMLPSI expected genetic gain per trait efficiency was 54.67%, whereas the average estimated OCMLPSI expected genetic gain per trait efficiency was 85% for all p values. Thus, the average of the estimated OCMLPSI accuracy associated with **d**′ = [3 -1] values was 35% higher than the average of the estimated DCMLPSI accuracy associated with **d**′ = [3-1] for the real dataset.

The results of the above four subsections indicate that the accuracy of both indices was higher when they predicted the selection response than when they estimated the expected genetic gain per trait. However, for the real data, the efficiency of the OCMLPSI when predicting the selection response and estimating the expected genetic gain per trait was higher than the DMLPSI efficiency when predicting the selection response and estimating the expected genetic gain per trait.

### OCMLPSI Efficiency vs. DCMLPSI Efficiency to Predict the Net Genetic Merit

Equation [10] is a tool for determining OCMLPSI efficiency vs. DCMLPSI efficiency when predicting the net genetic merit in percentage terms. The estimated average OCMLPSI efficiency to predict the net genetic merit in percentage terms is 100(97.604/90.019 - 1) = 8.426%, where 97.604 and 90.019 are the average of the estimated total OCMLPSI and DCMLPSI selection responses (Table 1) for all *p* values, respectively, and 8.426% is OCMLPSI efficiency with respect to DCMLPSI efficiency, in percentage terms, to predict the net genetic merit. Thus, for the Akbar et al. (1984) real dataset, the estimated average OCMLPSI efficiency was 8.426% higher than the estimated average DCMLPSI efficiency for predicting the net genetic merit.

### Simulated Data Estimated OCMLPSI and DCMLPSI Selection Response

For *p* = *q*_1_*q*_2_ = 0.01, 0.10, and 0.30, Table 3 presents the estimated OCMLPSI and DCMLPSI responses R^_1_ , R^_2_ , R^′_t_ = R^′_1_ + R^′_2_ and SCLPSI responses R^′_0.01_ , R^′_0.10_ , R^′_0.30_ ) for four simulated selection cycles in a two-stage breeding selection scheme. For *p* = 0.01, the average ofthe estimated total OCMLPSI selection responses (22.79) explained 99.80% of the average of the SCLPSI selection responses (22.84), whereas for *p* = 0.10 and 0.30, the average of the estimated total OCMLPSI selection responses (15.13 and 10.11, respectively) explained 100.60 and 101.81% of the average of the SCMLPSI selection response (15.04 and 9.93, respectively). Thus, for this dataset, the OCMLPSI and SCLPSI results were equivalent for all *p* values.

For *p* = 0.01, the average of the estimated total DCMLPSI selection responses (21.84) explained 95.62% of the average of the SCMLPSI selection responses (22.84), whereas for *p* = 0.10 and 0.30, the average of the estimated total DCMLPSI selection responses (14.43 and 9.49, respectively) explained 95.94 and 95.57% of the average of the SCMLPSI selection responses (15.04 and 9.93, respectively).

**Table 3.** Simulated data for total proportion retained (p)= q_1_q_2_ = 0.01, 0.10, and 0.30, and estimated optimum and decorrelated constrained multistage linear phenotypic selection indices (OCMLPSI and DCMLPSI, respectively) responses (*Ȓ*_1_, *Ȓ*_2_, and *Ȓ*_t_ = *Ȓ* +_2_) and single-stage constrained linear phenotypic selection index (SCLPSI) responses (*Ȓ*_0.01_, *Ȓ*_0.10_, and *Ȓ*_0.30_) for four simulated selection cycles in a two-stage breeding scheme.

Index	p = 0.01			OCMLPSI >p = 0.10			p = 0.30			SCLPSI>		
	Cycle	*Ȓ*_1_	*Ȓ*_2_	*Ȓ*_t_	*Ȓ*_1_	*Ȓ*_2_	*Ȓ*_t_	*Ȓ*_1_	*Ȓ*_2_	*Ȓ*_t_	*Ȓ*_0.01_	*Ȓ*_0.10_	*Ȓ*_030_
OCMLPSI	1	21.59	3.16	24.75	13.87	2.57	16.44	8.75	2.24	10.99	24.77	16.31	10.77
	2	20.39	3.09	23.47	12.99	2.6	15.59	8.18	2.24	10.42	23.54	15.50	10.24
	3	17.75	3.48	21.23	11.28	2.81	14.09	7.09	2.32	9.41	21.39	14.08	9.30
4	18.95	2.73	21.68	12.21	2.19	14.4	7.70	1.93	9.63	21.68	14.28	9.43
	Avg.	19.67	3.12	22.79	12.59	2.54	15.13	7.93	2.18	10.11	22.84	15.04	9.93
DCMLPSI	1	21.59	2.08	23.68	15.25	0.47	15.72	10.15	0.20	10.35	24.77	16.31	10.77
	2	20.39	2.12	22.51	14.40	0.48	14.88	9.58	0.20	9.78	23.54	15.50	10.24
	3	18.17	2.27	20.44	12.57	0.80	13.36	8.43	0.33	8.76	21.39	14.08	9.30
	4	18.95	1.77	20.72	13.38	0.40	13.78	8.91	0.17	9.07	21.68	14.28	9.43
Avg.	19.78	2.06	21.84	13.90	0.54	14.43	9.27	0.22	9.49	22.84	15.04	9.93

The results in this section indicate that although the average of the total OCMLPSI selection response, for all *p* values, overestimated the average of the SCLPSI by 0.73%, the average of the total DCMLPSI selection response, for all *p* values, underestimated the average of the SCLPSI by 4.30%. Thus, for this simulated dataset, the OCMLPSI was the best predictor of the net genetic merit, and its accuracy when predicting the selection response was higher than the DMLPSI accuracy for predicting the selection response.

### Estimated OMLPSI and DMLPSI Expected Genetic Gains per Trait

Table 4 presents the estimated OCMLPSI and DCMLPSI expected genetic gains per trait (E^′_1_ , **E^**′_2_ , and **E^**′_t_ = **E^**′_1_ + E^′_2_) for four simulated selection cycles and *p* = *q*_1_*q*_2_ = 0.30 in a two-stage context. Each E^′_t_ value was associated with the mean values of traits *T*_1_, *T*_2_, *T*_3_, and *T*_4_. In addition, in both indices, traits *T*_1_ and *T*_2_ were constrained by vector **d**′ = [5-2] values. The average of the estimated total OCMLPSIE^′_t_ values associated with traits *T*_1_ and *T*_2_ (5.76 and -2.30, respectively) overestimated the **d**′ = [5 -2] values by 15.20%. However, the average of the estimated total DCMLPSI E^′_t_ values associated with traits *T*_1_ and *T*_2_ (5.05 and -2.02, respectively) overestimated the **d**’ = [5 -2] values by only 1.0%. Nevertheless, note that at Stage 2, the averages of the estimated total DCMLPSI expected genetic gains per trait associated with traits *T*_1_, *T*_2_, and *T*_3_ (0.02, -0.01, and 0.0, respectively) were practically null. This means that DCMLPSI efficiency occurred at Stage 1, when restriction matrix **S**′_(*i*_ was null [**S**′_(*i* - 1)*i*_ = 0] and b^′_1_ = β^′_1_. Thus, for this dataset, the average of the estimated total DCMLPSI expected genetic gains per trait was more efficient for predicting the **d**′ = [5 -2] values than the OCMLPSI, but the highest DCMLPSI efficiency occurred at Stage 1, when b^′_1_ = β^′_1_ and the estimated standard deviations of OCMLPSI and DCMLPSI values were the same.

**Table 4.** Estimated optimum and decorrelated constrained multistage linear phenotypic selection indices (OCMLPSI and DCMLPSI, respectively) expected genetic gains per trait (E^) and (Ê′_1_, Ê′_2_, and Ê′_t_ = Ê′_1_, Ê′_2_) for Stages 1 and 2 in four simulated selection cycles with total proportion retained (*p* = *q*_1_*q*_2_ = 0.30 Traits *T*_1_ and *T*_2_ were constrained with vector d’ (a vector of constraints or predetermined proportional gains per trait imposed by the breeder) = [5 -2] values on the two indices.

Stage 1 Ê′_1_					Stage 2 Ê′_2_					Ê′_t_ = Ê′_1_, Ê′_2_				
Index	Cycle	*T*^1^	*T*^2^	*T*^3^	*T*^4^	*T*^1^	*T*^2^	*T*^3^	*T*^4^	*T*^1^	*T*^2^	*T*^3^	*T*^4^
OCMLPSI	1	4.55	−1.82	2.08	0.31	1.49	−0.59	0.66	0.54	6.03	−2.41	2.73	0.85
	2	4.30	−1.72	1.87	0.29	1.39	−0.56	0.60	0.59	5.69	−2.28	2.47	0.88
	3	3.87	−1.55	1.58	0.09	1.48	−0.59	0.57	0.64	5.36	−2.14	2.15	0.73
	4	4.50	−1.80	1.26	0.14	1.44	−0.58	0.40	0.43	5.94	−2.38	1.67	0.56
Avg.	4.31	−1.72	1.70	0.21	1.45	−0.58	0.56	0.55	5.76	−2.30	2.25	0.76
DCMLPSI	1	5.28	−2.11	2.41	0.35	0.01	−0.01	0.00	0.18	5.29	−2.12	2.41	0.53
	2	5.04	−2.02	2.19	0.34	0.01	0.00	0.00	0.18	5.05	−2.02	2.20	0.52
	3	4.60	−1.84	1.87	0.11	0.04	−0.01	0.00	0.28	4.64	−1.86	1.87	0.39
	4	5.21	−2.08	1.46	0.16	0.00	0.00	0.00	0.16	5.21	−2.08	1.46	0.32
	Avg.	5.03	−2.01	1.98	0.24	0.02	−0.01	0.00	0.20	5.05	−2.02	1.98	0.44

We also estimated the total expected genetic gains per trait of both indices for *p* = 0.01, 0.10, and 0.20 (data not shown); however, in all cases, those values were higher than the **d**′ = [5 -2] values. For example, for *p* = 0.10, the averages of the estimated total OCMLPSI and DCMLPSI expected genetic gains per trait associated with **d**’ = [5 -2] were 8.78 and -3.51, and 7.58 and -3.03, respectively.

The difference between the OCMLPSI and DCMLPSI expected genetic gains per trait is due to the different number of genotypes used to estimate the parameters. That is, in the real dataset, the number of genotypes was 3330, but in the simulated data, the number of genotypes was only 500, which represents only 15% of the size of the genotypes used in the real dataset to estimate the parameters of the indices. This means that the number of genotypes used to estimate the indices’ parameters was an important factor for both indices in the real and simulated data.

The results of the real and simulated datasets indicated that the OCMLPSI is the most efficient index for predicting the net genetic merit, and its accuracy when predicting the selection response and estimating the expected genetic gain per trait was higher than DCMLPSI accuracy when predicting the selection response and estimating the expected genetic gain per trait.

### Normality Test for the Estimated OCMLPSI and DCMLPSI Values at Stage 2

**Fig. 3:**
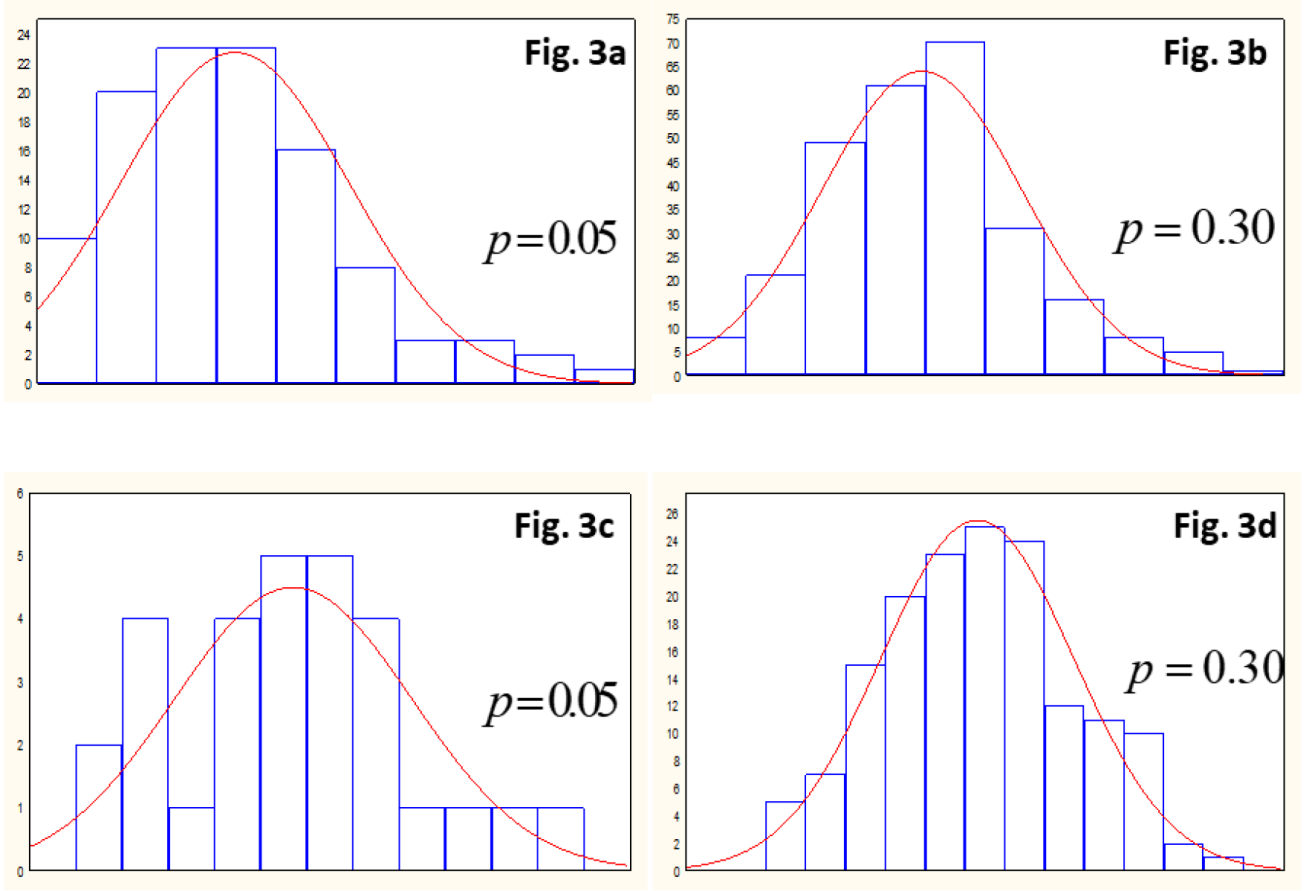
Histograms of the estimated optimum and decorrelated constrained multistage linear phenotypic selection index (OCMLPSI and DCMLPSI, respectively) values at Stage 2 for simulated dataset in Cycle 2, and the fixed total proportion retained (p) = **q**_1_q_2_ = 0.05 and 0.30when the number of genotypes was 110 (Fig. 3a) and 270 (Fig. 3b) for OCMLPSI, and 30 (Fig. 3c) and 155 (Fig. 3d) for DCMLPSI.

We used the simulated dataset in Cycle 2 to test the normality assumption of the estimated OCMLPSI and DCMLPSI values at Sage 2. In Cycle 1, the number of genotypes was 500. For *p* = *q*_1_*q*_2_ = 0.05 and 0.30, the *q*_1_ values for OCMLPSI were 0.22 and 0.55, whereas those values for DCMLPSI were 0.06 and 0.31, respectively. Then, at Stage 2, (0.2)(500) = 110 and (0.55)(500) = 270 were the number of genotypes for OCMLPSI, whereas for DCMLPSI, the number of genotypes were (0.06)(500) = 30 and (0.31)(500) = 155. We used these last numbers of genotypes to construct histograms (Fig. 3) of the estimated OCMLPSI and DCMLPSI values at Stage 2.

According to the histograms constructed for the estimated OCMLPSI values, when the number of genotypes changed from 110 (Fig. 3a) to 270 (Fig. 3b), the estimated OCMLPSI values were closer to the normal distribution. The same was true for the estimated DCMLPSI values (Fig. 3c and 3d).

We describe now the Shapiro-Wilk and Kolmogorov- Smirnov normality test results of the estimated OCMLPSI and DCMLPSI values at Stage 2 (Cycle 2) when the number of genotypes was 110 and 270 for OCMLPSI, and 30 and 155 for DCMLPSI. With the simulated dataset, we tested the null hypothesis that the estimated OCMLPSI and DCMLPSI values at Stage 2 have normal distribution.

The statistical value of the Shapiro-Wilk test should be close to 1.0 to accept the null hypothesis, whereas the statistic value of the Kolmogorov-Smirnov test should be close to 0.0 to accept the null hypothesis (Rani Das and Rahmatullah Imon, 2016). In the present case, for the values associated with OCMLPSI (110 and 270), the statistic values of the Shapiro-Wilk were 0.958 and 0.989, whereas the statistic values of the Kolmogorov-Smirnov were 0.080 and 0.044, respectively. Thus, we believe that for the estimated OCMLPSI values, the null hypothesis was true. In a similar manner, for the values associated with DCMLGSI (30 and 155), the statistic values of the Shapiro-Wilk were 0.967 and 0.991, whereas the statistic values of the Kolmogorov-Smirnov were 0.094 and 0.029, respectively. We again accept that the estimated DCMLPSI values approach the normal distribution.

## DISCUSSION

### Criteria Used to Evaluate the Relative Efficiency of the Indices

A criterion used to evaluate OCMLPSI efficiency vs. DCMLPSI efficiency when predicting the net genetic merit was that the estimated total OCMLPSI and DCMLPSI selection response must be lower than or equal to the single-stage estimated OCLPSI selection response. Additional criteria were the ratio of the OCMLPSI selection response over the DCMLPSI selection response and the estimated expected genetic gain per trait or multitrait selection response. The estimated total selection response of both indices predicted the mean value of the net genetic merit in the progeny population, whereas the estimated expected genetic gain values indicated how close the estimated mean values of the traits are to the predetermined proportional gains (or constraints) imposed by the breeder in each selection cycle. Both parameters are good criteria for comparing the efficiency of the indices, depending on the method used to estimate the vector of coefficients of each index.

### Selection Intensities

The selection intensities (*k*_1_ and *k*_2_) of both indices had three main parts: the proportions retained (*q*_1_ and *q*_2_), the truncation points (*u*_1_ and *u*_2_) and the height of the ordinate of the normal curve $\left[z\left(u_{1}\right)=e^{-0.5u_{1}^{2}}/\sqrt{2\pi }\;and\;z\left(u_{2}\right)=e^{-0.5u_{2}^{2}}/\sqrt{2\pi }\right]$ (Fig. 1). We obtained the *k*_1_ and *k*_2_ values for OCMLPSI with Eq. [A6] (Appendix B) method and with the Xu and Muir (1992) method for DCMLPSI. Both approaches were associated with the maximum total selection response R^′_t_ = k_1_Q^′_I1_ + k_2_Q^′_I2_) value, and the values of *k*_1_ and *k*_2_ were affected by the method used to obtain their values at Stages 1 and 2. When the *p* values changed from 0.05 to 0.30, the *u*_1_ and *u*_2_ values decreased, the *q*_1_ and *q*_2_ values increased and the k_1_ and k_2_ values decreased in both indices, as we would expect.

Equation [A6] (Appendix B) to obtain the OCMLPSI selection intensities in a two-stage context was proposed by Cerón-Rojas et al. (2019). These authors compared their results with the results of Saxton (1983), who used a numerical integration method to obtain truncation points, proportion retained, and selection intensities in a two-stage context. Saxton (1983) applied a two-stage selection scheme in two ways: first, by selecting three traits and then two traits; and second, by first selecting the last two traits and later the first three traits. Under the first scheme, Saxton (1983) found that the estimated total selection response overestimated the single-stage LPSI response by 3.8%, but under the second, he found that the estimated total selection response overestimated the single-stage LPSI response by only 1.5%. These results were very similar to the results obtained by Cerón-Rojas et al. (2019) when they used real data. This mains that, at least in a two-stage context, Equation [A6] was a good method to obtain the truncation points, proportion retained, and selection intensities.

### Number of Restrictions Imposed on the Indices

The OCMLPSI solved the OMLPSI equations subject to the restriction that the covariance between the OCMLPSI and some linear combinations of the genotypes involved be equal to a vector of predetermined proportional gains (or constraints) imposed by the breeder. However, in addition to the latter restriction, the DCMLPSI imposed the restriction that the covariance between DCMLPSI values at different stages be zero. The latter restriction decreased DCMLPSI efficiency after Stage 1, and as a result, its selection response and expected genetic gain were lower than the OCMLPSI selection response and expected genetic gain for the real and simulated datasets at Stage 2. Xu and Muir (1991, 1992) and Xie and Xu (1997) indicated that the loss of DCMLPSI efficiency after Stage 1 is justified because their method for obtaining the selection intensities and total responses gives the breeder the opportunity to implement an unlimited number of selection stages, which would otherwise be very difficult or impossible to do. At Stage 1, when the additional DCMLPSI restriction was null, the DCMLPSI and OCMLPSI vectors of coefficients were the same, as we would expect. Incidentally, this corroborated that both indices were applications of the SCLPSI to the multistage context.

According to Xu and Muir (1991,1992), the restriction that made the covariance between DCMLPSI values at different stages be zero is similar to the Kempthorne and Nordskog (1959) restriction imposed on the expected genetic gain per trait, which makes some traits not change their mean values while the rest of the trait means remain without restrictions. In effect, the DCMLPSI used a projector matrix (e.g., (**K**_*Di*_) to project the OMLPSI vector of coefficients (δ_i_) into a space smaller than the original space of δ_**i**_, whereas Kempthorne and Nordskog (1959) used a projector matrix to project the single-stage LPSI vector of coefficients into a space smaller than the original space of the LPSI vector of coefficients. The reduction of the space into which the Kempthorne and Nordskog (1959) matrix projects the LPSI vector of coefficients is equal to the number of zeros that appears in the expected genetic gain per trait, and the selection response and accuracy decrease as the number of restrictions increases (Cerón-Rojas and Crossa, 2018, Chapter 3). Nevertheless, it is not clear whether under the Xu and Muir (1992) restrictions the expected genetic gain per trait, the selection response, and the accuracy decrease as the number of stages increases. If this is true, the Xu and Muir (1992) method could not give the breeder the opportunity to implement an unlimited number of stages, because the expected genetic gain per trait, the selection response, and the accuracy will decrease as the number of stages increases and soon would be null.

In the DMLPSI context, Xie et al. (1997) compared the estimated single-stage LPSI selection response with the estimated DMLPSI selection response for two and three stages and found that at Stages 2 and 3, the estimated total DMLPSI selection response explained only 92 and 87%, respectively, of the estimated LPSI selection response. That is, at Stage 3, the estimated total DMLPSI selection response was lower (5%) than at Stage 2.

### Another Way of Writing the OCMLPSI and DCMLPSI Vectors of Coefficients

We wrote the OCMLPSI and DCMLPSI vectors of coefficients (β_*i*_ = **k**_Oi_ and **b**_i_ = **k**_Oi_δ_i_, respectively) as a projection of the OMLPSI vector of coefficients (**δ**_i_ = **Q**_ii_^-1^**A**_i_**w**) into a space that is perpendicular to the space generated by the columns of matrix **M**_i_(**V**_i_) made by the projector matrices **K**_*Oi*_^2^ and K_*Di*_ which are idempotent (**K**_*Oi*_ = **K**_*Oi*_^2^ and **K**_*Di*_ = **K***_Di_*^2^). This is the simplest way of writing the OCMLP SI and DCMLPSI vectors of coefficients. However, there is another way of writing the OCMLPSI and DCMLPSI vectors of coefficients based on the Tallis (1985) approach.

The Tallis (1985) approach requires a proportionality constant which, according to Itoh and Yamada (1987), represents the regression coefficient of the net genetic merit (H = **w**′**g**)on **Q**_*ii*_^-1^**V**_*i*_(**V**′_*i*_**P**^-1^**V**_*i*_)^-1^**d**_0_, where d_0_ is the DCMLPSI vector of predetermined restrictions. There are some problems associated with the proportionality constant. For example, if the proportionality constant is positive, it is appropriate for the DCMLPSI (OCMLPSI) vector of coefficients, and there is no problem; however, if the proportionality constant is negative, the indices will move the population means in the opposite direction to the predetermined desired direction.

### Another Constrained Multistage Index

Xie and Xu (1997) developed a constrained multistage selection index as an extension of the DMLPSI developed by Xu and Muir (1992) based on the Tallis (1962) index. Using the Akbar et al. (1984) real data, we found that the Xie and Xu (1997) index was not optimum. The average of the estimated Xie and Xu (1997) selection response for p = 0.05, 0.10, 0.20, and 0.30 explained only 68.55% of the one-stage SCLPSI, whereas the average of the estimated total OCMLPSI and DCMLPSI index selection responses explained 97.60 and 90%, respectively, of the estimated SCLPSI. Similarly, the estimated total Xie and Xu (1997) expected genetic gain values per trait explained only 10.17% of the vector d’ = [3 -1] values for both stages. These results indicated that, in effect, the Xie and Xu (1997) index is not optimum and breeders should not use it.

Cerón-Rojas and Crossa (2018) applied the OCMLPSI to the Hicks et al. (1998) dataset, but they used the Young (1964) method to obtain the selection intensities for two stages; thus, their results were approximations because the Young (1964) method overestimated the selection intensities (see Ceron-Rojas et al., 2019, for details).

### The Multivariate Normality Assumption of Both Indices

The multivariate normality assumption of the estimated OCMLPSI (DCMLPSI) values was the basis for developing the OCMLPSI (DCMLPSI) theory. Under this assumption, the total OCMLPSI selection response and expected genetic gain per trait for two or more stages, is the sum of each response and expected genetic gain per trait obtained at each stage. We corroborated the normality assumption at Stage 2 using histograms and normality tests. When at Stage 1 the number of genotypes was 500 and the total proportion retained was 5 or 30%, at Stage 2 the estimated OCMLPSI values approach the normal distribution in a similar manner as the estimated DCMLPSI values. These results indicate that the correlations between the estimated OCMLPSI values do not affect the normality distribution of the estimated OCMLPSI values, at least for the simulated dataset. This means that when the size of the population at Stage 1 is high (e.g., 500 or more), the correlations between the estimated OCMLPSI values cannot affect the normality distribution of the estimated OCMLPSI values in a two-stage context.

## CONCLUSIONS

We described the OCMLPSI and DCMLPSI theory and evaluated it in a two-stage context. Based on the estimated total selection response and the total expected genetic gain per trait of each index, we determined their efficiency using a real and a simulated dataset. We found that the OCMLPSI is the most efficient index for predicting the net genetic merit, and its accuracy for predicting the selection response and estimating the expected genetic gain per trait was higher than DCMLPSI accuracy for predicting the selection response and estimating the expected genetic gain per trait. Thus, breeders should use the OCMLPSI when making a selection, not the DCMLPSI.

## Abbreviations

BW: body weight
DCMLPSI: decorrelated constrained multistage linear phenotypic selection index
DMLPSI: unconstrained decorrelated multistage linear phenotypic selection index
EW: egg weight
LPSI: single-stage linear phenotypic selection index
OCMLPSI: optimum constrained multistage linear phenotypic selection index
OMLPSI: unconstrained optimum multistage linear phenotypic selection index
NMV: multivariate normal distribution
QTL: quantitative trait locus
REML: restricted maximum likelihood
RL: rate of lay
SCLPSI: single-stage constrained linear phenotypic selection index
SM: age at sexual maturity.

## APPENDIX A The Phenotypic Model to Estimate the Variance Components

In this work, we estimated matrices **P** and **G** (Eq. [1a]) using restricted maximum likelihood (REML) because this estimation method does not require a specific design or balanced data and can be used to estimate genetic and residual variance and covariance in any arbitrary pedigree of individuals. In addition, the expectation and maximization algorithm allows computing the REML for the variance components (Lynch and Walsh, 1998, Chapter 27; Cerón-Rojas and Crossa, 2018, Chapter 2).

Let

$$y_{q}=1\mu_{q}{\mathrm{Zg}}_{q}+e_{q}$$

be the phenotypic model where **y**_q_ is a g x 1(g=the number of genotypes in the population) vector of phenotypic averages, which has multivariate normal distribution (NMV) with mean 1µ*_q_* and covariance matrix **V**_q_; 1 is a g X 1 vector of ones, µ*_q_* is the mean of the qth trait, **Z** is an identity matrix g x g; **g**_q_ ~ NMV(**0**,**A**σ^2^_e__q_) is a vector of true breeding values, and ~ NMV(**0**,**I**σ^2^_e__q_) is a g X 1 vector of residuals. Matrix **A** denotes the numerical relationship matrix between individuals (Lynch and Walsh, 1998), and **V**_q_ = **A**σ^2^_g__q_ + **I**σ^2^_e__q_. We estimated and σ^2^_g__q_ and σ^2^_e__q_ in the absence of dominance and epistatic effects.

### Estimating Matrices G and P using the Expectation and Maximization Algorithm

The expectation and maximization algorithm allows computing the REML for the variance components σ^2^_g__q_and σ^2^_e__q_ by iterating the following equations:

[A1] $$\sigma_{g_{q}}^{2\left(n+1\right)}=\sigma_{g_{q}}^{2\left(n\right)}+\frac{{\left[\sigma_{g_{q}}^{2\left(n\right)}\right]}^{2}}{g}\times \left\{y_{q}^{\prime }\left[T^{\left(n\right)}{\mathrm{AT}}^{\left(n\right)}\right]y_{q}-\mathrm{tr}\left[T^{\left(n\right)}A\right]\right\}$$

and

[A2] $$\sigma_{e_{q}}^{2\left(n+1\right)}=\sigma_{e_{q}}^{2\left(n\right)}+\frac{{\left[\sigma_{e_{q}}^{2\left(n\right)}\right]}^{2}}{g}\times \left\{y_{q}^{\prime }\left[T^{\left(n\right)}T^{\left(n\right)}\right]y_{q}-\mathrm{tr}\left[T^{\left(n\right)}\right]\right\}$$

where, after *n* iterations, σ^2^_g_^2(n+1^ and σ^2^_e__q_^2(n+1)^ are the estimated variance components ofσ^2^_g__q_ and σ^2^_e__q_ respectively. In Eq. [A1] and [A2], tr(.) denotes the trace of the matrices within parentheses; **T** = **V**_q_^-1^ - **V**_q_^-1^**1**(**1**′**V**_q_^-1^**1**)**1**′**V**_q_^-1^ and **V**_q__-1_ is the inverse of matrix **V**_q_ = **A**σ^2^_g__q_ + **I**σ^2^_e__q_. In **T**^(n)^, **V**_q_^-1(n)^, is the inverse of matrix σ^2(n)^_γq_ + **I**σ^2(n)^_e__q_.

The additive genetic and residual covariances between the observations of the *q*th and ith traits, **y**_*q*_ and **y**_*i*_ (σ_g_q,i_ , and σ_e__q,i_q, *i* = 1, 2, *t*), can be estimated with REML by adapting Eq. [A1] and [A2] as follows. The variance of the sum of **y**_*q*_ and **y**_*i*_ can be written as Var(**y**_i_ + **y**_q_) = **V**_i_ + **V**_q_ + **2C***_iq_*^′^_iq_ where **V**_*i*_ = **A**σ*gi*^2^ + *I*σ_e__i_^2^ is the variance of **y**_*q*_; and **V**_*q*_ = **A**σ_g*q*_^2^ + **I**σ^2^_e_*_q_* is the variance of y_q_; in addition, 2**C***_iq_* = 2**A**σ*_giq + 2**I**σ_**_eiq_* = 2Cov(**y**, **y**_*q*_) is the covariance of **y**_*q*_ and **y***i*_ and σ*_giq_* and **σ***_eiq_* are the additive and residual covariances, respectively, associated with the covariance of **y***_q_* and **y***_i_*. Thus, one way of estimating is using the following equation: σ*_giq_* **σ*_eiq_***

[A3] $$0.5\mathrm{Var}\left(y_{i}+y_{q}\right)-0.5\mathrm{Var}\left(y_{i}\right)-0.5\mathrm{Var}\left(y_{q}\right)$$

for which Eq. [A1] and [A2] can be used.

### Estimating the OCMLPSI and DCMLPSI Selection Response and Expected Genetic Gain per Trait at Stage *i*

By Eq. [A1] to [A3], the estimates of matrices **P** and **G** can be denoted as **P^** and **G^** , and the estimated OCMLPSI and DCMLPSI selection responses (Eq. [8a] and [8b]) at Stage *i* are

[A4a] $${\hat{R}}_{Oi}=k_{Oi}\sqrt{{\hat{\beta }}_{i}^{\prime }{\hat{Q}}_{ii}{\hat{\beta }}_{i}}$$

and

[A4b] $${\hat{R}}_{Di}=k_{Di}\sqrt{{\hat{b}}_{i}^{\prime }{\hat{Q}}_{ii}{\hat{b}}_{i}}$$

whereas the estimated OCMLPSI and DCMLPSI expected genetic gains per trait (Eq. [9a] and [9b]) at Stage *i* are

[A5a] $${\hat{E}}_{Oi}=k_{Oi}\frac{{\hat{A}}_{i}^{\prime }{\hat{\beta }}_{i}}{\sqrt{{\hat{\beta }}_{i}^{\prime }{\hat{Q}}_{ii}{\hat{\beta }}_{i}}}$$

and

[A5b] $${\hat{E}}_{Di}=k_{Di}\frac{{\hat{A}}_{i}^{\prime }{\hat{b}}_{i}}{\sqrt{{\hat{b}}_{i}^{\prime }{\hat{Q}}_{ii}{\hat{b}}_{i}}}$$

respectively.

## APPENDIX B The OCMLPSI Selection Intensity for Two Stages

We describe a method to obtain the OCMLPSI selection intensity for fixed total proportion *p* = *q_1_q*_2_, where *q*_1_ and *q*_2_ are the proportions of individuals selected at Stage 1 and 2, respectively. This is because Cerón-Rojas et al. (2019) found that the Cochran (1951) and Young (1964) method overestimates the selection intensity. We obtained the DCMLPSI selection intensity according to the Xu and Muir (1992) method.

Let *I*_1_ = β^′^_1_x_1_ and *I*_2_ = β^′^_2_y be the OCMLPSI at Stages 1 and 2, respectively, and assume that the indices have bivariate normal distribution. Let *I*_1_ and *I*_2_ be transformed as *u*_1_ = (*I*_1_ - μ_*I*__1_)/σ_I_^1^ and *u*_2_ = (*I*_1_ - μ_*I*__2_)/σ_*I*_^2^ with mean zero and variance 1.0, where μ_*I*__1_ and μ_*I*__2_ are the means, whereas σ_*I*__1_ and σ_*I*__2_ are the standard deviations of *I*_1_ and *I*_2_, respectively. The selected population has bivariate left truncated normal distribution with probability density function *h*(*u*_1_, _u__2_) = *f*(*u*_1_, *u*_2_)/*p*, where *p* = *q*_1_*q*_2_,

$$f\left(u_{1}+u_{2}\right)=\frac{1}{2\pi \sqrt{1-\rho_{12}^{2}}}\exp\left[-\frac{1}{2\left(1-\rho_{12}^{2}\right)}\left(u_{1}^{2}+u_{2}^{2}-2\rho_{12}u_{1}u_{1}\right)\right]$$

and ρ_12_ is the correlation between *u*_1_ and *u*_2_ (Young, 1964; Cerón-Rojas and Crossa 2018, Chapters 2 and 9).

Consider the transformations (Springer, 1979) $\nu_{2}=\left(u_{2}-\rho_{12}u_{1}\right)/\sqrt{1-\rho_{12}^{2}}$, with Jacobian **j**, where

$$j^{-1}=\left|\begin{array}{cc} \frac{\partial v_{1}}{\partial u_{1}} & \frac{\partial v_{1}}{\partial u_{2}}\\ \frac{\partial v_{2}}{\partial u_{1}} & \frac{\partial v_{2}}{\partial u_{2}} \end{array}\right|=\left|\begin{array}{cc} 1 & 0\\ \frac{-\rho_{12}}{\sqrt{1-\rho_{12}^{2}}} & \frac{1}{\sqrt{1-\rho_{12}^{2}}} \end{array}\right|=\frac{1}{\sqrt{1-\rho_{12}^{2}}}$$

where |o| denotes the determinant function and ∂ denotes the partial derivatives of *v*_1_ and *v*_2_ with respect to *u*_1_ and *u*_2_. Thus,

$$v_{1}^{2}+v_{2}^{2}=\frac{u_{1}^{2}+u_{2}^{2}-2\rho_{12}u_{1}u_{2}}{\left(1-\rho_{12}^{2}\right)}$$

and

$$g\left(v_{1},v_{2}\right)=\left|j\right|f\left(u_{1},u_{2}\right)=\left(\frac{1}{\sqrt{2\pi }}e^{-0.5v_{1}^{2}}\right)\left(\frac{1}{\sqrt{2\pi }}e^{-0.5v_{2}^{2}}\right)$$

The transformations indicate that variables *v*_1_ and *v*_2_ are independent, each with a standard normal distribution.

Variables *v*_1_ and *v*_2_ are associated with the truncation points as $u_{2}=v_{2}\;\sqrt{1-\rho_{12}^{2}}+\rho_{12}v_{1}$. This means that *u*_1_ and *u*_2_ values should be obtained in two steps. First, we obtained the values of *v*_1_ and *v*_2_ from two independent standard normal distributions; then, we obtained the values of $u_{2}=v_{2}\;\sqrt{1-\rho_{12}^{2}}+\rho_{12}v_{1,\;}q_{1}$, and *q*_2_ finally, we obtained the OCMLPSI selection intensity at Stages 1 and 2 as

[A6] $$k_{1}=\frac{z\left(u_{1}\right)}{q_{1}}\mathrm{and}\;k_{2}=\frac{z\left(u_{2}\right)}{q_{2}}$$

respectively, where $z\left(u_{1}\right)=e^{-0.5u_{1}^{2}}/\sqrt{2\pi }\;and\;z\left(u_{2}\right)=e^{-0.5u_{2}^{2}}/\sqrt{2\pi }$ were the height of the ordinate of the normal curve at the lowest values of *u*_1_ and *u*_2_ retained, whereas *q*_1_ and *q*_2_ are the proportions of the population of animals or plants selected at each stage (Fig. 2). Equation [A6] values should be associated with the maximum *R*_t_ = *R*_1_ + *R*_2_ value (Fig. 2), where *R*_1_ = *k*_1_σ*_I_*_1_ and *R*_2_ = *k*_2_σ*_I_*_2_ are the selection responses, whereas σ*I*_1_ and σ*I*_2_ are the standard deviations of the variance of *I*_1_ and *I*_2_ at Stages 1 and 2, respectively.
